# Determinants of psychological distress among individuals who are aware of their HIV serostatus in South Africa: findings from the 2017 national HIV prevalence, incidence, behavior, and communication survey

**DOI:** 10.3389/fpubh.2024.1387878

**Published:** 2024-05-23

**Authors:** Noloyiso Vondo, Musawenkosi Mabaso, Thembelihle Ginyana, Lesiba Malope, Sizulu Moyo, Nompumelelo Zungu, Olive Shisana

**Affiliations:** ^1^Public Health, Societies, and Belonging Division, Human Sciences Research Council, Pretoria, South Africa; ^2^School of Nursing and Public Health and Family Medicine, University of Cape Town, Cape Town, South Africa; ^3^Nursing and Public Health, University of KwaZulu Natal, Durban, South Africa; ^4^Department of Psychiatry and Mental Health, University of Cape Town, Cape Town, South Africa; ^5^EB Consulting, Pty, Ltd., Cape Town, South Africa

**Keywords:** psychological distress, HIV serostatus, South Africa, HIV negative, HIV positive

## Abstract

**Introduction:**

Psychological distress is a growing public health challenge among people living with HIV. This study investigated the prevalence of psychological distress among individuals who know their HIV positive or negative serostatus in South Africa using 2017 data from a nationwide cross-sectional household-based population survey.

**Methods:**

The data for this secondary analysis was collected using a multi-stage stratified cluster randomized sampling design. Multivariable backward stepwise generalized linear regression models were fitted to determine factors associated with psychological distress as measured by the Kessler Scale (K10) among HIV-positive and HIV-negative individuals who know their serostatus in South Africa.

**Results:**

Of 18,662 participants, psychological distress was 27.4% (95% CI: 25.3–29.7) among those HIV-positive and 20.1% (95% C: 18.8–21.4) among those HIV-negative. The odds of psychological distress were significantly higher among HIV-positive individuals who rated their health as fair/poor [AOR = 1.22 (95% CI: 1.09–1.35), *p* < 0.001], and the odds were lower among those residing in rural formal/farm areas [AOR = 0.85 (95% CI: 0.78–0.93), *p* < 0.001], and those with tertiary education level [AOR = 0.88 (95% CI: 0.78–0.99), *p* = 0.033]. The odds of psychological distress in HIV-negative individuals were significantly higher among females than males [AOR = 1.09 (95% CI: 1.05–1.14), *p* < 0.001], high-risk alcohol drinkers [AOR = 1.26 (95% CI: 1.02–1.57), *p* = 0.035] and hazardous alcohol drinkers [AOR = 1.09 (95% CI: 1.01–1.18), *p* = 0.028] than abstainers and those who rated their health as fair/poor rather than excellent/good [AOR = 1.18 (95% CI: 1.10–1.26), *p* < 0.001].

**Conclusion:**

The study underscores the importance of addressing, alcohol misuse and socio-structural inequalities linked to gender and race-based disparities, such as low educational attainment and unemployment, as critical factors associated with psychological distress in the study population.

## Introduction

Mental health disorders such as psychological distress, as measured by depressive and anxiety symptoms, represent a growing public health challenge and are more prevalent in people living with HIV (PLHIV) than in the general population (1, 2). Globally, the pooled prevalence of psychological distress among PLHIV was estimated to be 28%, and between 10 and 34% in Sub-Saharan Africa (2). In South Africa, previous estimates of psychological distress among PLHIV vary substantially with higher rates ranging between 20 and 30% (2–4). These estimates highlight the significant impact of mental health challenges on PLWH.

Several studies have suggested a complex and bidirectional relationship between HIV infection and psychological distress. HIV infection can be a consequence of risky behaviors that are associated with poor mental health, or pre-existing mental illness that alters one’s behaviors (2–4). HIV diagnosis can also be unbearable leading to emotional and mental stress experienced due to shock, shame, fear and worry (2, 4–8). In addition, stigma, discrimination, and negative attitudes experienced by PLHIV contribute to psychological distress in this population group (5–8).

Despite significant advances that have been made in HIV treatment, coping with the reality of living long with a chronic illness like HIV remains a challenge (9). Psychological distress has been associated with treatment interruptions and sub-optimal adherence to ART, increasing the potential for virological failure, poor disease outcomes, and further spread of HIV (8, 9). Consequently, the goal of ending the HIV epidemic may not be achieved without addressing the significant mental health challenge among PLHIV (9). The general challenge is that a substantial proportion of PLHIV have existing mental disorders that are likely to go undetected and therefore untreated (7, 10).

PLHIV who are vulnerable to mental health conditions often face individual, structural, and social challenges to accessing and adhering to HIV prevention and treatment modalities (8). Evidence shows that increased vulnerability to poor mental health outcomes among PLHIV vary with socio-demographic and structural factors such as age, race, sex, poverty, low education, and unemployment (7, 8). Comorbid communicable diseases and noncommunicable diseases also contribute to poorer physical and mental health outcomes among PLHIV (8, 9). Experiences of enacted, perceived and internalized stigma among PLHIV contribute to psychological distress and exacerbate barriers to accessing adequate and sustained healthcare (5–8).

Evidence shows that contextual forces intersect with demographic, social, and structural factors in vulnerable communities to shape mental health outcomes among PLHIV (7–9, 11, 12). However, gaps in national level evidence and heterogeneous findings perpetuates an unintegrated health care system in many countries in sub-Saharan Africa (13). Consequently, this study is premised on linkages between persistent health disparities and multiple risk factors across varied contexts (14, 15). Guided by the theory of fundamental causes which recognizes multiple disease outcomes to a health condition; the influence of multiple risk factors; that resources can be used to avoid risks or to minimize the consequences of disease; and that the association between a fundamental cause and health are reproduced over time in the absence of intervening mechanism (16). This theoretical framework guided the analysis of the inter-relationships between HIV and psychological distress and associated background and socio-demographic factors.

Large-scale population-based studies are also needed especially in a high HIV-prevalence country like South Africa to influence policy change, and designing of service delivery models for implementation of integrated strategy at national level. This study investigated the prevalence of psychological distress and associated factors among HIV-positive and HIV-negative youth and adults 15 years and older who are aware of their serostatus using 2017 nationally representative population-based household survey.

## Materials and methods

### Data source and study sample

This secondary analysis used data from the 2017 South African national HIV prevalence, incidence, behavior, and communication survey (17). This is a nationally representative population-based household survey conducted using a multi-stage stratified random cluster sampling design described in detail elsewhere (17). Briefly, a total of 1,000 small area layers (SALs) were drawn from a national master sampling frame of 86,000 SALs released by Statistics South Africa in 2001 and updated in 2011. The selection of SALs was stratified by province, locality type (urban areas, rural informal and formal areas), race group, and sex. A systematic probability sample of 15 visiting points/household was drawn from each of the 1,000 SAL, yielding a total of 15,000 visiting points/household targeted for the survey.

A household questionnaire and three age-appropriate questionnaires were administered to consenting individuals. Given by parents/guardians and assent by the participant. The interview instruments solicited information on socio-demographic characteristics, HIV-related knowledge, attitudes, and behaviors. Dried blood spots (DBS) specimens were used to collect blood from consenting individuals for HIV antibody testing using an algorithm with three different enzyme immune assays (EIAs). All samples testing HIV positive during the first two EIAs (Roche Elecys HIV Ag/Ab assay, Roche Diagnostics, Mannheim, Germany, and Genescreen Ultra HIV Ag/Ab assay, Bio-Rad Laboratories, California, United States) were subjected to a nucleic acid amplification test (COBAS AmpliPrep/Cobas Taqman HIV-1 Qualitative Test, v2.0, Roche Molecular Systems, New Jersey, United States) for the final interpretation of test results. Testing for exposure to antiretroviral drugs (ARVs) in HIV-positive specimens was performed using High-Performance Liquid Chromatography (HPLC) coupled with Tandem Mass Spectrometry.

The current study is based on a sub-sample of youth and adults 15 years and older who tested for HIV and were aware of their serostatus and had responded to the 10-item scale used as a brief screening tool to measure psychological distress.

### Ethical consideration

The survey protocol was approved by the Human Sciences Research Council (HSRC) Research Ethics Committee (REC: 4/18/11/15), and the Division of Global HIV and TB (DGHT) and the Centre for Global Health (CHG) of the Centre for Disease Control and Prevention (CDC).

### Measures

#### Dependent variable

The primary outcome variable in this study is psychological distress derived from Kessler Psychological Distress Scale (K10) (18). This scale has been validated among low- and middle-income countries, including South Africa (18–20). The K10 scale measures anxiety and depressive symptoms by asking: During the previous 30 days how often did you feel: tired out for no good reason; so nervous that nothing could calm you down; hopeless, restless or fidgety, so restless that you could not sit still; depressed; that everything was an effort; so sad that nothing could cheer you up; worthless? The frequency with which each one of these items were felt was captured on a 5-point Likert scale (1 = never, 2 = rarely, 3 = some of the time, 4 = most of the time, 5 = all of the time). The raw scores are then summed, and a total score is used to indicate that respondents are likely to be well (score below 20), experiencing mild (score 20–24), moderate (score 25–29) or severe (score 30 and above) psychological distress. For this analysis, due to the small numbers in each of the five categories, the scores were dichotomized into a binary outcome: - those who scored <20 (absence of psychological distress = 0) and those who scored ≤20 (presence of psychological distress = 1).

#### Independent variables

Explanatory variables include socio-demographic variables:- age categories in years (15–24, 25–49, 50 years and older), sex (male, female), race (African, other), marital status (married, never married), educational level (no education/primary, secondary, tertiary), employment status (unemployed, employed), and locality type (urban, rural farm, rural informal), risky behavior and health-related variables:- experiences of physical intimate partner violence (no, yes), alcohol use measured using the AUDIT score (0 = abstainers; 1–7 = low-risk drinkers; 8–19 = high-risk drinkers; 20+ = hazardous drinking), and self-rated health (excellent/good, fair/poor).

### Statistical analysis

Descriptive statistics were used to summarize the study sample and sociodemographic, behavioral, and HIV-related variables by psychological distress stratified by HIV status. Multivariable backward stepwise generalized linear regression models were fitted to determine the factors associated with psychological distress among HIV-positive and HIV-negative individuals who were aware of their serostatus. Adjusted Odds Ratios (AOR) with 95% Confidence Intervals (CI) and *p*-value ≤0.05 were used to determine the direction of the relationship and the level of statistical significance. All analysis was conducted using STATA Statistical Software Release 15.0 (Stata Corporation, College Station, TX, United States).

## Results

### Sample characteristics

The study sample consisted of 18,662 participants, 4,401 tested positive for HIV status, and 14,261 were HIV-negative. Table 1 shows that the majority of HIV-positive participants were aged 25 to 49. Overall, most participants were female, Black African, had never been married, had secondary-level education, were unemployed, and resided in urban areas. The majority of all participants reported experiences of IPV, most abstained from alcohol, and had excellent/good self-rated health.

**Table 1:** Characteristics of the study sample, HIV-positive and HIV-negative youth and adults 15 years and older (*n* = 18,662).

Variables	Study sample		HIV-positive		HIV-negative	
	Total	%	n	%	n	%
Age categories (years)						
15–24	4,275	20.5	492	9.2	3,783	23.8
25–49	9,772	59	3,153	76.4	6,619	54.0
50 and older	4,615	20.5	756	14.4	3,859	22.2
Sex						
Male	6,484	42.3	1,043	31.1	5,441	45.5
Female	12,178	57.7	3,358	68.9	8,820	54.5
Race groups						
African	15,223	83.3	4,224	96.5	10,999	79.6
Other	3,439	16.7	177	3.5	3,262	20.4
Marital status						
Married	5,149	32.4	806	23.5	4,343	35.0
Never married	11,738	67.6	3,181	76.5	8,557	65.0
Education level						
No education/Primary	2,949	16.9	843	20.6	2,106	15.8
Secondary	9,961	68.7	2,620	73.4	7,341	67.3
Tertiary	1,601	14.3	221	6.0	1,380	17.0
Employment status						
Unemployed	12,762	64.7	3,100	68.0	9,662	63.8
Employed	5,690	35.3	1,264	32.0	4,426	36.2
Locality type						
Urban	10,183	66.3	2064	61.3	8,119	67.8
Rural informal (tribal areas)	6,688	28.8	1865	33.8	4,823	27.3
Rural (farms)	1791	4.9	472	4.9	1,319	4.9
AUDIT score						
Abstainers	12,774	67.5	3,289	74.9	9,485	65.3
Low-risk drinkers (1–7)	2,974	21.1	565	16.5	2,409	22.5
High-risk drinkers (8–19)	1,326	9.9	259	7.6	1,067	10.6
Hazardous drinkers (20+)	207	1.5	40	1.0	167	1.7
Physical IPV						
No	3,738	88.1	893	24.9	2,845	18.0
Yes	481	11.9	135	33.0	346	26.6
Self-rated health						
Excellent/good	15,132	80.9	3,331	75.9	11,801	82.3
Fair/poor	3,518	19.1	1,067	24.1	2,451	17.7

Not all sub-totals add to the overall totals due to non-response and missing data; IPV, intimate partner violence; AUDIT, Alcohol Use Disorder Identification Test.

### Prevalence of psychological distress

Of 18,662 participants, psychological distress was significantly higher (*p* < 0.001) among HIV-positive individuals who were aware of their status [27.4% (95% CI: 25.3–29.7)] compared to HIV-negative individuals who were aware of their status [20.1% (95% C: 18.8–21.4)]. Table 2 shows the distribution of psychological distress by sample characteristics stratified by HIV status. Among HIV-positive individuals who were aware of their serostatus, psychological distress was significantly higher among those aged 50 years and older, the unemployed and those who rated their health as fair/poor. Among HIV-negative individuals who were aware of their serostatus, psychological distress was significantly higher among females, Black Africans, those with no education or with primary level education, the unemployed, those who reported experiences of physical IPV, hazardous alcohol drinkers and those who rated their health as fair/poor.

**Table 2:** Prevalence of psychological distress by sample characteristics and HIV serostatus.

Variables	HIV Positive				HIV negative			
	n	%	95% CI	*p*-value	Neg	%	95% CI	*p*-value
Age (years)				0.053				0.171
15–24	492	23.8	18.3–30.4		3,783	18.8	16.7–21.0	
25–49	3,153	26.9	24.5–29.5		6,619	20.0	18.6–21.6	
50 and older	756	32.4	27.9–37.3		3,859	21.5	19.3–23.9	
Sex				0.333				<0.001
Male	1,043	25.9	22.3–29.8		5,441	16.2	14.7–17.9	
Female	3,358	28.1	25.5–30.8		8,820	23.3	21.6–25.0	
Race groups				0.119				<0.001
African	4,224	27.7	25.5–30.0		10,999	21.5	20.1–22.9	
Other	177	20.4	13.6–29.6		3,262	14.5	12.2–17.2	
Marital status				0.304				0.324
Married	806	24.8	20.5–29.6		4,343	18.8	16.8–20.9	
Never married	3,181	27.5	25.0–30.2		8,557	20	18.5–21.6	
Education level				0.065				0.002
No education/Primary	843	31.4	27.2–36.0		2,106	25.6	22.8–28.5	
Secondary	2,620	27.1	24.4–30.1		7,341	19.3	17.9–20.9	
Tertiary	221	21.1	14.4–29.8		1,380	18.3	15.0–22.1	
Employment status				<0.001				<0.001
Unemployed	3,100	30.3	27.7–33.0		9,662	22.1	20.6–23.6	
Employed	1,264	21.7	18.5–25.2		4,426	16.4	14.7–18.4	
Locality type				0.363				0.189
Urban	2064	28	25.1–31.2		8,119	20.9	19.3–22.5	
Rural informal/tribal areas	1865	27.1	23.8–30.6		4,823	18.8	16.5–21.3	
Rural formal/farm areas	472	21.8	16.0–29.0		1,319	16.3	11.2–23.0	
AUDIT score				0.274				<0.001
Abstainers	3,289	27.6	25.1–30.3		9,485	20	18.5–21.6	
Low-risk drinkers (1–7)	565	22.9	18.5–28.0		2,409	16.2	13.9–18.7	
High-risk drinkers (8–19)	259	29.8	22.2–38.8		1,067	23.2	19.9–26.9	
Hazardous drinkers (20+)	40	21.7	9.7–41.8		167	45.2	35.0–55.8	
Physical IPV				0.112				0.007
No	893	24.9	20.8–29.4		2,845	17.9	15.9–20.2	
Yes	135	33	23.7–43.8		346	26.6	20.3–34.0	
Self-rated health				<0.001				<0.001
Excellent/good	3,331	22.1	19.9–24.6		11,801	16.8	15.6–18.1	
Fair/poor	1,067	44.1	39.1–49.2		2,451	35.2	32.4–38.1	

Not all sub-totals add to the overall totals due to non-response and missing data. IPV, intimate partner violence; AUDIT, Alcohol Use Disorder Identification Test.

### Factors associated with psychological distress

**Figure 1:**
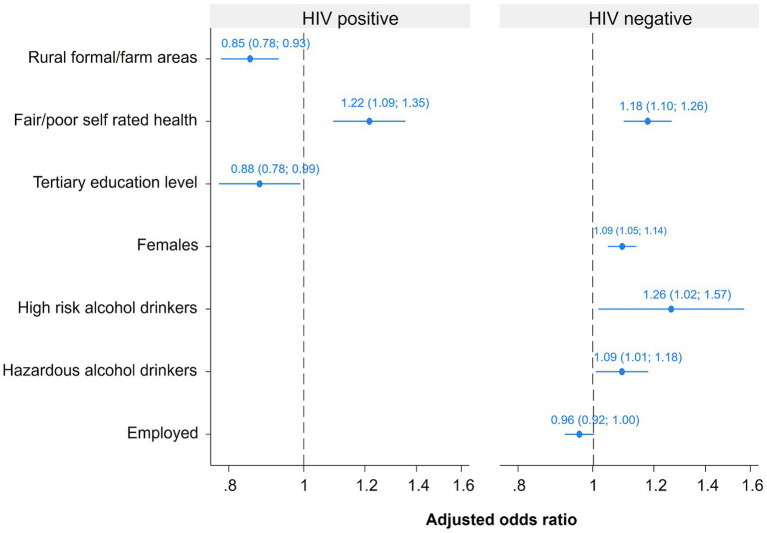
Multivariate models of factors associated with psychological distress among HIV-positive and HIV-negative youth and adults 15 years and older.

Figure 1 shows a multivariate logistic regression model of factors associated with psychological distress among HIV-positive and HIV-negative persons who are aware of their serostatus. The odds of psychological distress in HIV-positive individuals were significantly higher among those who rated their health as fair/poor than excellent/good [AOR = 1.22 (95% CI: 1.09–1.35), *p* < 0.001]. In this group, the odds of psychological distress were lower among those who reside in rural formal/farm areas than in urban areas [AOR = 0.85 (95% CI: 0.78–0.93), *p* < 0.001], and those with tertiary education level than no education or primary education level [AOR = 0.88 (95% CI: 0.78–0.99), *p* = 0.033]. The odds of psychological distress in HIV-negative individuals were significantly higher among females than males [AOR = 1.09 (95% CI: 1.05–1.14), *p* < 0.001], high-risk alcohol drinkers [AOR = 1.26 (95% CI: 1.02–1.57), *p* = 0.035] and hazardous alcohol drinkers [AOR = 1.09 (95% CI: 1.01–1.18), *p* = 0.028] than abstainers and those who rated their health as fair/poor rather than excellent/good [AOR = 1.18 (95% CI: 1.10–1.26), *p* < 0.001].

## Discussion

This nationally representative survey revealed that the prevalence of psychological distress was considerably higher among HIV-positive youth and adults 15 years older who knew their status compared to their HIV-negative counterparts who also knew their status. A meta-analysis of studies comparing HIV-positive and HIV-negative samples showed that major depressive disorder occurred nearly twice as often among HIV-positive than HIV-negative patients (21). This is similar to global estimates and within the range of prevalence reported in sub-Saharan Africa among PLHIV (1, 3, 22). Overall, 24.7% levels of psychological distress were reported among PLHIV in this study. This is lower than 34.7 and 32.6% reported in other studies in South Africa (3, 4, 22). In other studies, heterogeneity in the prevalence of psychological distress has been partially attributed to different sampling methods, differences in the timing of the studies assessment tools and partly due to different population characteristics (1, 3, 22).

In the present study, the prevalence of psychological distress among HIV-positive individuals was significantly higher for those 50 years and older. This is similar to other studies that revealed that high levels of psychological distress stress were associated with older adults living with HIV (3, 4). Aging is associated with a decline in physical and cognitive abilities, which increases the risk of developing multiple chronic illnesses, including mental health disorders (23–25), and this is of particularly high concern among PLHIV (25). Therefore, older people with HIV need to get linked to HIV care and access mental health and other support services to help them stay healthy and remain engaged in HIV care (26).

The prevalence of psychological distress among HIV-negative individuals was significantly higher among females, Black Africans, the unemployed and those who reported experiences of physical IPV. In other studies, the observed differences have been associated with social stressors linked to gender and racial differences in socioeconomic resources (27). It has also been shown that gender plays an important role in explaining the incidence of intimate partner violence (28, 29). The final model suggests that unemployment was marginally associated with psychological distress in HIV-negative individuals. HIV-negative females remained associated with psychological distress in the final models. These observations suggest a continued need to address resource inequality related to gender, race, and unemployment to reduce mental health disparities among South Africans.

The prevalence of psychological distress among HIV-negative individuals was also higher among high-risk alcohol drinkers, and this variable was also retained in the final model. Alcohol use disorder often co-occurs with other mental health disorders, either simultaneously or sequentially (30). The most common mental health conditions that co-occur with alcohol use disorders are depression and anxiety (31). The findings reiterate the importance of harmful alcohol use as an urgent public health priority in South Africa. This highlights the need for comprehensive interventions targeting co-occurring alcohol use disorders and psychological distress.

Furthermore, those who rated their health as fair or poor had a higher prevalence of psychological distress, regardless of whether they were HIV-positive or HIV-negative. The final model also suggests that self-rated health may be an important predictor of poor mental health outcomes in both HIV-positive and HIV-negative individuals. Self-rated health has been used as a reliable, quick assessment tool for population health monitoring (32, 33). Other findings also suggest that measuring people’s subjective health can be a useful tool for assessing the risk of mental health issues in HIV-related care and support programs (8). However, evaluating this measure for use in such services is important.

In addition, the model suggests that psychological distress is associated with no education or low educational attainment among HIV-positive individuals, as observed in other studies (34, 35). On the contrary others found no association between educational status and general psychological distress (36). Lack of education or low educational attainment are indicators of poverty. Research indicates that those living with HIV in impoverished communities are disproportionately affected by mental health issues (37, 38). These observations underscore the importance of integrated HIV mental health interventions linked with poverty alleviation strategies in resource-limited settings (37, 38).

Furthermore, the final model indicates that psychological distress is significantly associated with living in urban areas among HIV-positive individuals. In South Africa, the HIV burden varies geographically and is highest in urban settings (13, 39). Evidence shows that the high levels of psychological distress may be linked to the persistently high level of HIV prevalence in urban settings (40, 41). This can be attributed to the collective hardship of poverty and unemployment in urban areas, combined with the high burden of HIV, and these factors can lead to increased psychological distress (13, 42). These observations suggest that the government should continue supporting policies that improve situational circumstances and address the unmet social and mental health needs, especially in highly HIV-burdened communities.

### Limitations

The cross-sectional design limits the study, so causal relationships between dependent and independent cannot be determined. The study also relied on self-report and is prone to recall and social desirability bias. There may also be other confounding or unmeasured factors that were not accounted for in the secondary analysis. Wide confidence intervals were observed in the results of some of the regression analyses, indicating the uncertainty of effect sizes due to low counts for observed variables. Notwithstanding these limitations, the study was based on a nationally representative sample and can be generalized to HIV-positive and HIV-negative youth and adults 15 years and older in the country.

### Public health implications

The study findings of considerably high levels of psychological distress among PLHIV are crucial for communities of practice, which comprise social workers, psychologists, practitioners, and clinicians. Thus, rather than adopting disjointed approaches, mental health among PLHIV ought to be at the forefront of public health engagement across a range of platforms, including conferences, workshops, seminars, and professional training programs. Public health practitioners must be equipped with the knowledge and resources necessary to enhance their comprehension of the causes, consequences, and expressions of psychological distress in PLHIV. Ultimately, this will enable public health professionals to develop and implement tailored community-based interventions to support and care for PLHIV in the fight against mental disorders.

## Conclusion

The study found that HIV-positive individuals who knew their status were more likely to experience psychological distress compared to those who were HIV-negative. The results showed that increasing age, low educational attainment and residing in urban settings increase the risk of psychological distress among HIV-positive individuals. In addition, psychological distress is influenced by gender and race-based disparities, including unemployment. Maladaptive behaviors such as alcohol abuse and IPV also contribute to psychological distress. Overall, the study emphasizes the importance of addressing alcohol use disorders and socio-structural inequalities like poverty, low educational attainment, and unemployment as critical factors associated with psychological distress in the study population. Based on the current findings appropriate screening and treatment for psychological distress should be considered for comprehensive HIV care. Therefore, integrating mental health services with HIV care and support program is necessary for reducing negative life events associated with psychological distress among PLHIV. Further research is needed on the impact of psychosocial and HIV-related factors on mental health of HIV-seropositive individual especially given the scaling up of ART.

## Data availability statement

The original contributions presented in the study are included in the article/supplementary material, further inquiries can be directed to the corresponding author.

## Ethics statement

The studies involving humans were approved by the Human Sciences Research Council Ethics Committee. The studies were conducted in accordance with the local legislation and institutional requirements. Written informed consent for participation in this study was provided by the participants’ legal guardians/next of kin.

## Author contributions

NV: Conceptualization, Formal analysis, Writing – original draft, Writing – review & editing. MM: Conceptualization, Formal analysis, Supervision, Writing – original draft, Writing – review & editing. TG: Writing – original draft, Writing – review & editing. LM: Writing – original draft, Writing – review & editing. SM: Funding acquisition, Writing – original draft, Writing – review & editing. NZ: Funding acquisition, Writing – original draft, Writing – review & editing. OS: Conceptualization, Writing – original draft, Writing – review & editing.
